# Strategies for Effective Capacity-Building in the Fight Against Commercial Tobacco

**DOI:** 10.5888/pcd21.230307

**Published:** 2024-05-23

**Authors:** Kimberly Caldwell, Ashley Hebert, Gregory Bolden

**Affiliations:** 1 The Center for Black Health & Equity, Durham, North Carolina

## Abstract

The Center for Black Health & Equity’s approach to addressing health inequities relies on the inherent ability within community-based organizations to respond to public health priorities while addressing the political and social determinants of health. By using Dr. Robert Robinson’s Community Development Model as a foundational framework, communities can address systemic barriers that impede optimal health outcomes. The model includes community engagement and mobilization activities that motivate communities to achieve equity-centered policy change and offers milestones that show progress made toward their goals and objectives. We operationalized the Community Development Model into the Community Capacity Building Curriculum to train community partners to form a multicultural coalition through asset mapping as a tool for community mobilization. This curriculum is both cost effective and efficient because it enables communities to address health disparities beyond tobacco control, such as food and nutrition, housing, and environmental issues. Coalitions are prepared to identify and make recommendations to address policies that perpetuate health disparities. Facing off against a powerful tobacco industry giant is challenging for small grassroots organizations advocating for stricter tobacco regulations and policies. Such organizations struggle for resources; however, their passion and dedication to the mission of saving Black lives can promote change.

**Summary**

**What is already known on this topic?**

For the past twenty years, tobacco control partnerships and coalitions have raised awareness of the harms of tobacco products through public health campaigns and cessation services.

**What is added by this report?**

Program planning requires a strategic approach, such as that offered through our Community Capacity Building Curriculum and RoadMap tool for ongoing community support.

**What are the implications for public health practice?**

Health justice is inherently linked to social justice. Our training series provides a detailed community-led action plan that accounts for each community’s history, culture, context, and geography to support the successful implementation of tobacco control programs.

## Introduction

The Center for Black Health & Equity (hereinafter, The Center) originally founded as the National African American Tobacco Prevention Network, develops new coalitions or identifies existing ones to build capacity within the Black community to advocate for policies promoting optimal health. For over 20 years, The Center has built networks across the country to drive meaningful change in diminishing health disparities and promoting health equity; it is one of the Centers for Disease Control and Prevention’s (CDC’s) 9 funded national networks in its National Networks Driving Action: Preventing Tobacco- and Cancer-Related Health Disparities by Building Equitable Communities program. The Center reduces health disparities as a grantee of the program by advancing commercial tobacco prevention with evidence-based strategies that address cancer-related illness among African Americans who experience tobacco- and cancer-related health disparities.

The Center strategically partners with community-based organizations, national partners, and state and local public health departments to ensure tobacco control activities and policies benefit the Black community. The health implications of equitable policies minimize the harm for people who are most affected by the problem. The outcome of these activities will benefit the community in the following ways: the government system will provide equitable protections to disparate populations; predatory marketing, density of tobacco-selling venues, and advertising in unprotected communities will be reduced; health care will benefit from improved and equitable cessation services and decreased chronic disease prevalence; smoking and overall tobacco use will be reduced in housing, especially multiunit housing with units that share ventilation systems; and school systems will see a reduction in student suspensions for violating tobacco-free school policies. Our primary focus has been educating the public and community leaders about the harmful effects of mentholated tobacco use and highlighting the availability of culturally tailored cessation services.

The purpose of this article is to provide learners — the public health community, community leaders, and the general public — with the tools of our Community Capacity Building Curriculum. This process will enrich learners with theoretical strategies that can be implemented in local and state community-based organizations. Once implemented in the community, these strategies will also reduce risk factors for chronic disease and improve quality of life among community members, especially those affected by tobacco-related illnesses. The learner will gain the fundamental process to engage community members to expand their outlook and build their leadership capacity skills, empowering them to work toward collective change through structured processes (1).

## Menthol and the Black Community

Big Tobacco’s infiltration into the Black community started before many of us were born (1), and its hold remains firm today. By centuries of forced enslavement in farming tobacco, covert marketing campaigns targeting Black Americans, and political tactics that pit Black influencers against public health progress, this infiltration has achieved Goliath-level proportions. Decades after our fight against Big Tobacco began, the world’s 4 largest tobacco companies (2) continue to cause chronic illnesses and death and severely hinder any progress in improving local, state, and national health, including disparate economic conditions.

Big Tobacco uses Black community leaders to lobby against policies and laws, such as a menthol ban, that would reduce smoking in Black communities. A Food and Drug Administration ban on the manufacturing, distribution, and wholesale, import, and retail sale of menthol cigarettes and little cigars would address health inequities caused by the industry’s targeting. Pending such a ban, state and local governments have taken action to prohibit the retail sale of flavored tobacco products, including menthol cigarettes. These policies are a big step forward for health equity and social justice in the US (3). Additionally, Big Tobacco uses preemption as a weapon to block the passage of local bans on menthol (1,4). With the knowledge of how important local control is to tobacco prevention efforts, the industry and its allies historically have used preemptive strategies to hamper smoke-free laws and influence youths’ access and tobacco retailer licensing policies (5,6).

## The Community Capacity Building Curriculum

Our Community Capacity Building Curriculum is based on Dr. Robert Robinson’s Community Development Model, an asset-based framework that builds on existing community strengths and capacity while developing infrastructure and competency to assess problems and implement solutions (7). The model offers the learner measurable milestones toward goals and objectives, making the process sustainable. Communities are empowered to replicate efforts beyond tobacco control, such as with food and nutrition, housing, and environmental issues. Communities that decide to mobilize and fight for themselves will also be prepared to identify and make recommendations for centering policies in equity.

The curriculum operationalizes the foundational framework of the Community Development Model (8) into modules and navigates essential stages — mobilization, assessment, planning, implementation, and evaluation — by using the RoadMap, a tool we developed for ongoing guidance (Figure). This model prioritizes community members, encouraging them to identify their unique needs and assets to develop home-grown solutions. From assessing the nuances of diverse community landscapes to celebrating hard-won victories, the curriculum addresses not only immediate issues, but cultivates a culture of self-reliance, pride, and long-term empowerment. Together, these frameworks are not merely theoretical constructs but dynamic tools for crafting a more equitable, healthy, and vibrant community. Through planning, implementation, and outcomes, the RoadMap helps advocacy groups and coalitions achieve measurable milestones, which ultimately lead to their desired policy, system, and environmental change. Communities identify needed assets (research, programs, leaders, organizations, networks) and if feasible, implement necessary development strategies.

**Figure:**
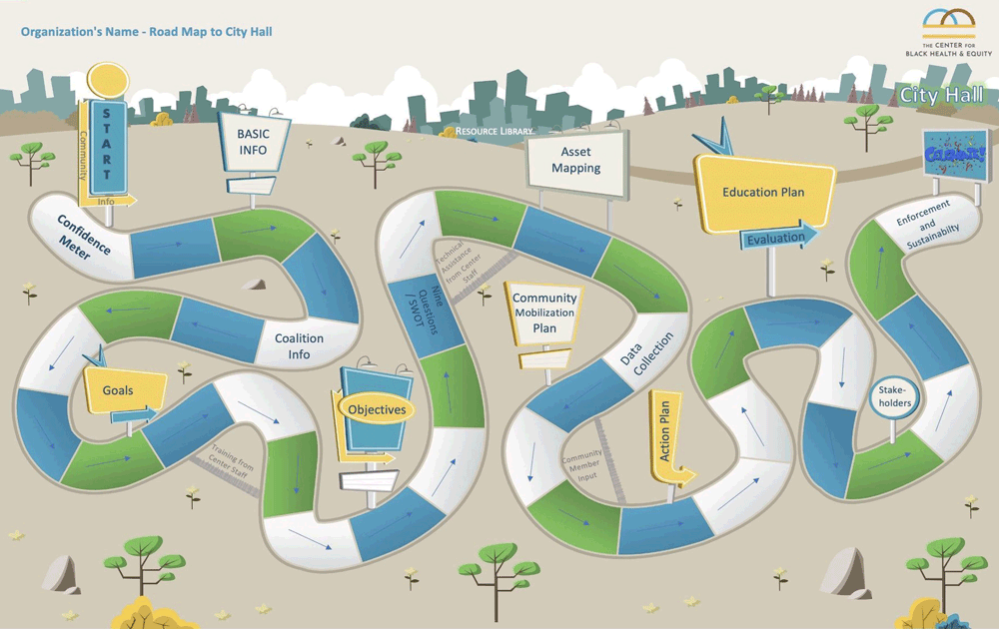
A visual representation of the RoadMap interactive tool of the Center for Black Health & Equity. The map is an interactive Excel file that houses all the elements of the Community Capacity Building Curriculum. The user can click on a segment, which links to an Excel cell that describes a particular tool of the curriculum, such as the Confidence Meter, Asset Mapping Tool, Community Mobilization Plan, and others. The RoadMap houses the coalition’s created assets throughout their completion of the series.

The Community Capacity Building Curriculum consists of 5 in-person or virtually led modules. Through the 5-module series, the curriculum facilitates building capacity in data collection, community mobilization, education campaigns, policy development, and ongoing evaluation improvement. The modules serve as a how-to guide for community mobilization against commercial tobacco.

### Module 1: History of tobacco, menthol, racism, and Black Americans

Understanding the history and context of commercial tobacco use and its disproportionate effect on Black and other communities of color is critical to identifying ongoing inequities and industry tactics. In the first module, an assessment of the history, context, culture, and geography of tobacco use helps coalitions understand the determinants of that community. At the end of Module 1, attendees will be able to

- Identify at least 3 examples of how the tobacco industry targets Black American communities with mentholated tobacco products
- Identify how the tobacco industry markets mentholated tobacco products to Black Americans, such as through advertisements, sales, and social media
- Understand the historic reasons why Black Americans have health disparities resulting from use of tobacco and mentholated tobacco
- Understand how commercial tobacco has shaped the development of the US
- Identify a minimum of 4 key strategies to reduce tobacco-related disparities in the Black American community

### Module 2: Coalition development and maintenance

The Center works to build capacity with established coalitions and to form new ones. Of utmost importance is the development of multicultural, multiethnic, multigenerational coalitions representative of the community. In this module, groups begin to organize by assessing resources and cultivating leaders.

Resources or assets can include individuals, organizations, institutions, buildings, landscapes, equipment — anything that can be used to improve a community’s quality of life. Building the capacity of leaders who are community assets creates opportunities for people affected by a problem to participate, build relationships, and influence changing the problem. Community leaders are often unknown outside the community because they lack the opportunity to be seen by outsiders. Identifying who these people are and working with them is important. By the end of Module 2, attendees will be able to

- Identify and understand the use of infrastructure- and capacity-building tools for developing the coalition
- Identify key skill sets required for advocacy work toward building community inclusiveness
- Determine the readiness and needs of the coalition to effectively advocate for a menthol flavor restriction ordinance
- Identify the key community assets required for a successful menthol ban advocacy campaign
- Identify subcommittees to lead specific tasks for building the coalition
- Name and brand the coalition

### Module 3: Action planning

Coalitions develop an action plan based on the RoadMap that leads to desired policy, system, and environmental change. The RoadMap is an interactive tool designed to guide development of an action plan to educate community members and other key players on the dangers of menthol and other flavored tobacco products and guide development of strategies to reduce tobacco-related disparities in their community. Attendees make contributions to the action plan by providing their understanding of the history, context, culture, and geography of their experiences with tobacco use, predatory marketing, and resource identification and by defining roles and responsibilities to lead activities recommended by attendees. This module also includes completion of The Democracy Center’s “Nine Questions for Strategic Advocacy Strategy Planning Framework” (9) and an analysis of strengths, weaknesses, opportunities, and threats related to policy, system, and environmental change. Answers to the questions are subject to change over time because the implementation of plans may require a pivot in strategies and activities. At this stage, the coalition will also develop skills in policy development, data collection, and most importantly, community mobilization. At the end of Module 3, attendees will be able to

- Develop the elements of an action plan (RoadMap) for their menthol flavor restriction project that consists of goals, measurable objectives, strategies, and activities
- Agree on the direction of the coalition and policy, system, or environmental change
- Identify strengths, weaknesses, opportunities, and challenges of the project
- Identify resources in hand and resources needed
- Develop outcome measures to determine success
- Prepare for the development of their RoadMap

National partners participate in these modules, leading or contributing to discussions and serving as subject matter experts when appropriate. The Center engages national partner organizations to assist in the capacity-building of coalitions to fight for themselves against systemic injustice. Organizations such as the African American Tobacco Control Leadership Council (AATCLC) assist with educating decision makers and key allies, the Public Health Law Center provides legal guidance and model policy language examples, and the CDC Office on Smoking and Health provides national data and visits the discussions at various points during the training process.

### Module 4: Education campaign training

Coalitions begin to develop their education campaigns tailored to the priority population of the geographic area the policy, system, or environmental change will affect. Here the coalitions are trained to develop messaging, imagery, and dissemination channels for the products developed. Emphasis is placed on earned media, social media, and paid media strategies. At the end of Module 4, attendees will be able to

- Develop messaging and imagery toward educating the community on the dangers of using menthol and other flavored tobacco products
- Develop a dissemination plan for the education campaign in the selected community

### Module 5: The RoadMap

Coalitions build, launch, and execute their RoadMap and action plans. At the end of Module 5, all attendees will be able to

- Develop the action plan RoadMap by plotting the key elements in a virtual tool designed to illustrate the plan to advocate for policy, system, and environmental change.

## Fighting Menthol with Community Mobilization

Community mobilization is the process of bringing together diverse interested individuals and groups to raise awareness of and demand for a shared goal, to assist in the delivery of resources and services, and to strengthen community participation for sustainability and self-reliance (10). When people from different parts of the community share a common goal and actively participate in both identifying needs and being part of the solution, that community is empowered to initiate and control its own development (10).

Detroit, Michigan, is a shining example of a community that uses its community assets to enact public health change. Minou Jones is the founder of Making It Count Community Development Corporation (https://www.umakeitcount.org), a nonprofit organization bringing positive change to underserved Detroit communities through a series of programs and services. She currently serves as chair of the Detroit–Wayne–Oakland Tobacco-Free Coalition (DWOTFC) and as a board member for Tobacco-Free Michigan. DWOTFC, a fully functional and funded organization, is a leading example of the successful implementation of our capacity-building curriculum. Upon completion of the curriculum steps and the RoadMap tool, she guides a multisector coalition of community groups and members working to change public policy in Michigan and ban flavored tobacco products in the state.

Minou Jones’s work has influenced policymakers in Detroit, where the city council responded to DWOTFC’s education efforts by passing a resolution that asks Michigan to ban menthol and other flavored commercial tobacco and eliminate outdated state preemption policies that prohibit cities from regulating flavored tobacco within their own borders. The resolution was approved by the city council without objection (11). It is now a key element of larger coalition education and advocacy efforts urging the state legislature to modernize preemption laws so city leaders have the authority to ban flavored tobacco and protect their residents.

For decades communities have mobilized against Big Tobacco by using a multicultural, multiethnic, and multigenerational approach. The US Department of Health and Human Services Community Preventive Services Task Force recommends using community mobilization along with supplementary measures, such as enforcing stricter local laws for retailers, actively ensuring compliance with sales laws, and educating retailers with enforcement, all backed by solid evidence of the effectiveness of these measures in reducing tobacco use by children and adolescents and access to tobacco products from commercial sources (12). These interventions were designed to influence the public to move toward positive change in their communities.

### An Example From the Field: San Francisco, California — Carol McGruder, African American Tobacco Control Leadership Council (AATCLC) Founding Member and Co-Chair

AATCLC is the leading public health education and advocacy organization in the US that is taking on Big Tobacco to save Black lives. The group has grown a coalition of national and local organizations. Its representatives have traveled across the country to educate communities and build grassroots infrastructure, and the organization has achieved major legislation ending the sale of menthol-flavored tobacco products in cities, counties, and states across the country. AATCLC has sued the Food and Drug Administration to prohibit the sale of menthol-flavored tobacco products throughout the US.

Carol McGruder:** “**I embrace accountability with grace, mercy, and patience with coalitions and communities. Over the course of my relationship with the [California] Department of Public Health, I have learned that you have to re-engage people and provide consistent messaging. I have advocated for opportunities to try a different way and support people along the process*.*

“We started the African American Tobacco Control Leadership Council (AATCLC) because funding for priority population leadership groups was cut. The Latinos, the Asians, and the LGBTQ groups (along with the AATCLC), were really the only ones who banded together and kept going. I think it's because of our nature of fighting, the way that we do this is so different than other groups because we have really had to fight for everything.

“Look at how Barack Obama got in and look at what happened. The pendulum swings back and forth and everything that we do as Black people and any gain we make, there's going to be forces pushing back. Whether it’s politically, health wise, etc. We see it hugely in the industry (Big Tobacco).”

Traditional evaluation for assessing program activities often fails to put community members or people as the center focus, and often overlooks the Black American lived experience. The Center measures success by its ability to get coalitions successfully through the series of modules. However, the curriculum as a whole has strengths and weakness that should be considered (Box).

Box **The Center for Black Health & Equity Evaluation of the Strengths and Weakness of the 5-Part Capacity Building Process**

**Table:** 

Strengths	Weaknesses
Community-led: The rate of success vastly improves when the community of focus is involved from the onset and not an afterthought.	Competing interests from national lobbying organizations can derail the process of the 5-part series of modules because of their top-down approach.
Training protocol: You must complete Module 1 before moving to Modules 2–5.	Timeline: Completion of the curriculum series can take up to 1 calendar year, often competing with legislative calendars and the available time of the coalition.
Training products: Each module results in a product that is loaded into the RoadMap. All products are results of the corresponding module.	Training format: Most trainings are delivered virtually. The attention to detail is higher in person because the trainer can observe in-person interactions and troubleshoot on the spot if additional in-person trainings are conducted.
Subcommittees: The subcommittees mirror the trainings that we have facilitated, and these subcommittee members put steps in place to move their action plan and RoadMap to success.	None
Visibility: The modules provide local organizations with additional visibility and exposure to other local and national organizations in the commercial tobacco control space while also giving them a sense of control and agency to choose their partner organizations.	Political environment: The political climate is assessed. The ideal level of engagement should include the state health department, the local coalition, national partners, and The Center. At times we do not have the ideal engagement because of the political climate. We have this in Detroit, and funding is from the state health department.

### Conclusion

The Center has combined its wealth of knowledge of Black history, tobacco industry tactics, health disparities, and racism to curate its Community Capacity Building Curriculum, a series of trainings that progressively move Black communities toward health justice through a lens of health equity. Based on our community development model, which includes community engagement and community competence (1), the process empowers Black communities to move toward policy identification and policy change. The Community Capacity Building Curriculum offers participants measurable milestones that allow community members to see their progress toward their goals and objectives. With each step in the process, measurable competencies are developed, implemented, and ultimately evaluated: plans for community mobilization for advocating for policy, systems, and environmental change; educating key decision makers and the overall community; and sustaining efforts for the long term. Importantly, the greatest outcome of the process is the growth and development that occurs among participants. These skills and competencies enable community members to take greater control of their own lives and, ultimately, address their own concerns, now and into the future. Lessons learned will enable communities to replicate efforts beyond tobacco control, in areas such as food and nutrition, housing, and environmental issues.

Through our community trainings, we have learned that education and awareness are necessary to move communities with limited experience to take advocacy action for tobacco control. Education and awareness can broaden local commitment to policy change by changing social norms in affected communities, such as the use of tobacco to manage stress. Local organizations with experience working in areas related to social justice and health equity were able to build on their expertise and credibility in their community as a result of working through the 5 modules of our Community Capacity Building Curriculum (12).

The Center is building the capacity of communities in Charleston, South Carolina; Cleveland, Ohio; New Orleans, Louisiana; Beckley, West Virginia; Grand Rapids, Michigan; Detroit, Michigan; Albany, Georgia; and Charlotte, North Carolina, to develop and implement action plans directed at local policies to restrict the sale of menthol and other flavored commercial tobacco products and eliminate exposure to secondhand smoke.

By marrying strategy with action, our curriculum aims to create an environment in which effective change is possible and sustainable. Our commitment to sustainable change is evidenced by our thorough planning to evaluate and document outcomes through the innovative RoadMap process, ensuring that the effect of our interventions is long-lasting and continually adapted based on community feedback. Through this multifaceted approach, our Capacity Building Curriculum seeks to transform the values, needs, and aspirations of local communities, thereby making the principles of equity and social justice tangible realities.
